# Physiological, biochemical, and metabolic changes in diploid and triploid watermelon leaves during flooding

**DOI:** 10.3389/fpls.2023.1108795

**Published:** 2023-03-09

**Authors:** Nan He, Muhammad Jawad Umer, Pingli Yuan, Weiwei Wang, Hongju Zhu, Xuqiang Lu, Yan xing, Chengsheng Gong, Raufa Batool, Xiaowu Sun, Wenge Liu

**Affiliations:** ^1^ Zhengzhou Fruit Research Institute, Chinese Academy of Agricultural Sciences, Zhengzhou, China; ^2^ Department of Horticulture, Hunan Agricultural University, Changsha, Hunan, China; ^3^ State Key Laboratory of Cotton Biology/Institute of Cotton Research, Chinese Academy of Agricultural Sciences (ICR, CAAS), Anyang, Henan, China; ^4^ State Key Laboratory for Biology of Plant Diseases and Insect Pests, Institute of Plant Protection, Chinese Academy of Agricultural Sciences, Beijing, China

**Keywords:** watermelon, diploid, triploid, flooding, abiotic stress

## Abstract

**Background:**

Flooding is a major stress factor impacting watermelon growth and production globally. Metabolites play a crucial role in coping with both biotic and abiotic stresses.

**Methods:**

In this study, diploid (2X) and triploid (3X) watermelons were investigated to determine their flooding tolerance mechanisms by examining physiological, biochemical, and metabolic changes at different stages. Metabolite quantification was done using UPLC-ESI-MS/MS and a total of 682 metabolites were detected.

**Results:**

The results showed that 2X watermelon leaves had lower chlorophyll content and fresh weights compared to 3X. The activities of antioxidants, such as superoxide dismutase (SOD), peroxidase (POD), and catalase (CAT), were higher in 3X than in 2X. 3X watermelon leaves showed lower O_2_ production rates, MDA, and hydrogen peroxide (H_2_O_2_) levels in response to flooding, while higher ethylene production was observed. 3X had higher levels of dehydrogenase activity (DHA) and ascorbic acid + dehydrogenase (AsA + DHA), but both 2X and 3X showed a significant decline in the AsA/DHA ratio at later stages of flooding. Among them, 4-guanidinobutyric acid (mws0567), an organic acid, may be a candidate metabolite responsible for flooding tolerance in watermelon and had higher expression levels in 3X watermelon, suggesting that triploid watermelon is more tolerant to flooding.

**Conclusion:**

This study provides insights into the response of 2X and 3X watermelon to flooding and the physiological, biochemical, and metabolic changes involved. It will serve as a foundation for future in-depth molecular and genetic studies on flooding response in watermelon.

## 1 Introduction

Flooding is a major factor limiting plants growth, development as well as production worldwide (Rao et al., 2016). Watermelon is widely grown globally, despite its vulnerability to flooding stress, particularly during its early development stage. Flooding has become a major challenge hindering watermelon production. Improving the waterlogging tolerance of watermelon is therefore a pressing concern that requires immediate attention.

Watermelon produces large edible fruits which provide important part of the human diet world over (Erickson et al., 2005). Watermelon is a highly valuable crop and contributes 2.56% to global vegetable production, according to data from FAO (http://faostat.fao.org). In 2020, China consumed over 70 million tons of watermelon, with a per capita consumption of over 50 kilograms (kg) (http://faostat.fao.org). Consumers prefer seedless watermelon cultivars for their high quality and premium price compared to seeded (2n) watermelon (Marr and Gast, 1991).

Flooding occurs due to torrential rains as well as dwindling soil draining (Voesenek et al., 2014; Zhang et al., 2016). Plants require oxygen to survive and perform metabolic processes, but when the soil becomes flooded, excessive water can reduce the availability of oxygen and cause significant damage. This can result in stunted root and shoot growth, as well as alterations in phenological and physiological processes. As a result, plants become more vulnerable and may struggle to gather the necessary nutrients to survive and perform photosynthesis (Boru et al., 2003). Moreover, flooding leads to lipid peroxidation which in turns cause an undue accretion of reactive oxygen species (ROS) (Hasanuzzaman et al., 2020). Reactive oxygen species **(**ROS) accumulation leads to cell death, and oxidative damage is due to the imbalance among antioxidant e production (AOX) and ROS (Hussain et al., 2019; Zafar et al., 2019). Increasing activity of defense-related enzymes such as peroxidase (POD) and catalase (CAT) can scavenge ROS in plants and improve plant’s resistance to stress (Meng et al., 2019; Noor et al., 2019). Guaiacol peroxidase (POX) and CAT convert H_2_O_2_ to H_2_O (Ahmad and Wani, 2014). Previous researches has already presented that higher ethylene contents due to flooding also effects root and shoot development (Khan et al., 2018), lower photosynthesis (Peltonen-Sainio and Rajala, 2001; Gommers et al., 2013). Biosynthesis of ethylene starts when methionine changes to S-adenosyl-L-methionine (SAM) *via* SAM synthetase. Next, ACC is formed *via* SAM that is catalyzed with 1-aminocyclopropane-1-carboxylic acid (ACC) synthase. The ACC subsequently oxidized to ethylene *via* ACC oxidase (Zhang et al., 2019).

Metabolomics research presents a unique opportunity to discover a significant number of crucial metabolites. Previous studies have utilized tolerant plant varieties to study the role of metabolites in enabling plants to withstand harsh environmental conditions. This approach is done with meticulous care, as the use of tolerant plant varieties provides valuable information about how plants respond metabolically to environmental stress (Lothier et al., 2020). Metabolites carries signals and move in intra and inter-cellular environments and are directly or in directly involved in the growth and development as well as defense (Evans, 2003). Plants respond to various stimuli at the transcriptional and translational levels, leading to changes at the cellular level. Gene expression may be altered in response to internal or external stimuli. Transcriptomics allows us to quantify and identify numerous responsive genes that may play a role in resistance or tolerance to environmental stress. The combination of different omics approaches can provide a deeper understanding of a plant’s response to flooding. Advanced techniques such as GCMS, LCMS, and NMR are effective in detecting various types of metabolites in plants in response to different stressors. Primary metabolites are involved in lipid production, sugar synthesis, and amino acid production, which have an impact on plant growth and development. The activities of primary metabolites can lead to the formation of secondary metabolites in response to stressors such as flooding, high temperatures, cold, drought, salt, and insect/pest attacks.

There is a significant amount of research currently being conducted to gain a deeper understanding of the molecular mechanisms involved in the response of plants to flooding. These studies aim to provide insight into the plant’s response mechanisms and enhance our knowledge of the effects of flooding on plants (Fukao and Xiong, 2013; Mustroph et al., 2015) and low-O_2_ sensing experience have been performed in Arabidopsis and then tested in barley to enhance its ability to tolerate low-O_2_ (Mendiondo et al., 2015). Scientists have identified SUBIA and SNORKL 1 and 2 in rice (Xu et al., 2006; Hattori et al., 2009). (Licausi et al., 2011; Sasidharan and Mustroph, 2011; He et al., 2022) that are responding effectively in low O_2_ and helps to understand its molecular mechanism. SUBIA and ENORKET 1 and 2 works with ERF-VII which is a Transcription factor (Sasidharan et al., 2013; Veen et al., 2013). Nevertheless, of reduced O_2_ sensing, signaling, and lower response networks, there remain breaches. Significant challenges exist within the comprehension and enhance root aeration, along with the management of lower-O_2_ metabolism, as well as recoveries after stress.

Currently, we are using 2X and 3X watermelon to examine the physiological and biochemical changes in response to flooding. For the first time, we are employing a metabolomics-based approach to identify metabolites that are associated with tolerance to flooding in watermelon leaves. Our study involves quantifying metabolites in the leaves of 2X and 3X watermelon using an UPLC-ESI-MS/MS system. Our results indicate that the 3X watermelon performed better than the 2X watermelon in response to flooding stress. This research will contribute to our understanding of the underlying mechanisms involved in watermelon’s response to flooding and provide information about the response of diploid and triploid watermelon to flooding.

## 2 Materials and methods

### 2.1 Plant materials and sowing details

In this experiment, the diploid variety Zh2X (Zhengzhou #3) and the triploid variety Zh3X (Zhengzhou #3) were used. The experiment was carried out at the research base in Zhengzhou fruit Research Institute of CAAS. Matured and uniform seeds were soaked in water for at least three hours at room temperature before sowing. The seeds were germinated at 35°C for 36 hours and then transferred to pots filled with substrate mixed with carbendazim fungicide. The experiment was conducted in March 2019 in a greenhouse located in Zhengzhou, Henan province, China. During a 12-hour light cycle, the temperature was maintained at 26°C and during a 12-hour dark cycle, the temperature was kept at 23°C.

Once the fourth true leaf had fully developed, the plants were subjected to flooding stress by submerging them in a water tank. The water level was maintained by adding water daily. Samples were collected and phenological observations were made 0, 3, 5, and 7 days after flooding with the 0-day samples serving as the control. Six plants were used per sample and counted as a replicate. In total, three biological replicates were collected, as detailed in **Supplementary Table 1**.

### 2.2 Estimating chlorophyll contents (CC) and plasma membrane permeability (PMP)

For estimating the CC of watermelon leaves under flooding, we used a SPAD-502 chlorophyll meter to record SPAD values (Konica Minolta, Tokyo, Japan). Moreover, for measuring the PMP we followed an already established protocol describes by Aly-Salama and Al-Mutawa (2009).

### 2.3 Estimation of reactive oxygen species (ROS) along with malondialdehyde (MDA) content

For the estimation of ${\text{O}}_{2^{-}}$ production rate an already established protocol was followed as described earlier by Pompeiano et al. (2019). For estimating the H_2_O_2_ content we used a kit “kit YX-W-A400” from (Sino Best Biological Technology Co., Ltd). For the measurement of MDA content, an already established protocol described by Wang et al. (2010) and Zhu et al. (2020) was used.

### 2.4 Estimation of antioxidant enzymes activities

Activities of peroxidase (POD), superoxide dismutase (SOD), as well as catalase (CAT) were measured by following the protocols described earlier by Wang et al. (2014) and Kosar et al. (2021). Contents of proline was estimated as described in a protocol by McGee et al. (2021).

### 2.5 Assessment of non-enzymatic antioxidants

Herein, 1g of frozen leaf samples were crushed and homogenized in a 5% cold meta-phosphoric acid followed by a 15 min centrifugation at 11,500 RPM and 4 °C. Further, the contents of ascorbate and glutathione were determined using the clear supernatant from step 1. To determine the Ascorbic acid +Dehydrogenases activity and Ascorbic acid contents, we followed the procedure as described by Zhang et al. (Zhang et al., 2012). The difference between AsA+DHA and AsA estimated the concentration of Dehydrogenases activity (DHA). Substantially, the oxidized glutathione (GSSG) as well as total glutathione (GSH+GSSG) levels were recorded by the method described by Nahar et al. (2015). GSH content was calculated by subtracting GSSG by total GSH.

### 2.6 Quantification of Ethylene

Leaves of watermelon under flooding were collected and put in a saturated NaCl. Test tubes were used for the collection of excreted gases by using the glass funnel. An air tight syringe was used to collect the collected gas samples and also for injecting into the machine for performing chromatography (GC-2010, Shimadzu) (Yamauchi et al., 2015).

### 2.7 Preparation od samples for extraction and widely targeted metabolic analysis

For the widely targeted metabolic analysis, root samples were collected 0, 3, 5, and 7 days after flooding and kept at -80°C. The freeze-dried samples were used for metabolite extraction using the method and machine conditions described by Wu et al. (2020) and He et al. (2022).

### 2.8 Principal component analysis (PCA) analysis

Principle component analyses (PCA) were acquired *via* an R-based statistical program prcomp (www.r-project.org) (3.50). Data were log2 converted before performing the PCA.

### 2.9 Hierarchical cluster analysis as well as the calculation of pearson correlation coefficients

The results of the sample and metabolites were presented using a Hierarchical Cluster Analysis (HCA). The Pearson Correlation Coefficient (PCC) between the samples was calculated using the cor function in R, and the relationship between the samples was visualized in the form of heatmaps using the heatmap package (version 1.0.13). The signal intensity of the metabolites was standardized using unit variance scaling and represented as a color scale in the HCA.

### 2.10 RNA isolation to perform quantitative real-time PCR

The collected frozen leaf samples were used to isolate RNA using the TreliefTM RNAprep Pure Plant Kit from TSINGKE, located in Beijing, China. The quality of the RNA was determined through electrophoresis on a 1.0% agarose gel. The Universal SYBR qPCR Master Mix from Vazyme Biotech Co., Ltd., located in Nanjing, China, was utilized following the methodology described previously by Yamauchi et al. (2015). *ClaActin* was used as the internal control. To perform RT-qPCR we used three biological replicates. The sequences of primers used are given as **Supplementary Table S2**.

### 2.11 Statistical analysis

The significance of the data was analyzed using one-way ANOVA, and the significant differences between the individual averages were calculated using the Least Significant Difference (LSD) method with a significance level of P < 0.05 and P < 0.01. The results were plotted using Microsoft Excel (Khoshnamvand et al., 2020). Heatmaps were generated by using TBtools software (Chen et al., 2020).

## 3 Results

### 3.1 Fluctuations in chlorophyll content as well as other growth indices

**Figure 1:**
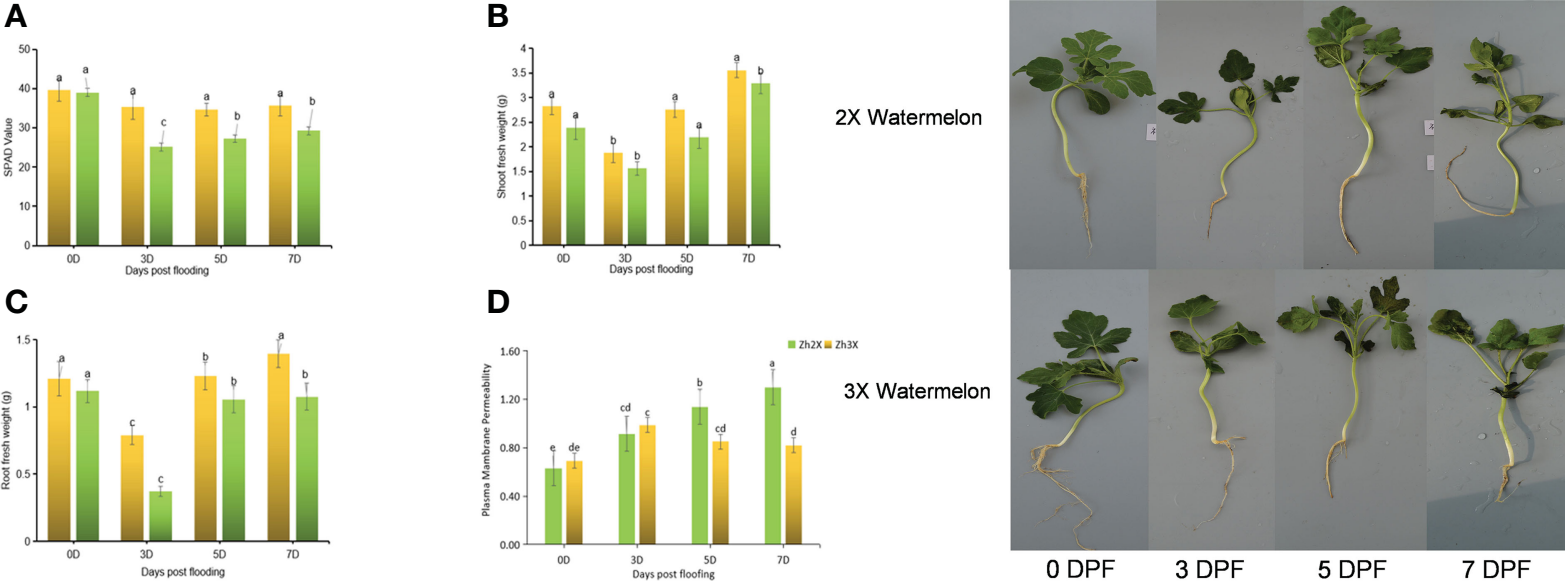
Fluctuations in Chlorophyll Content as well as other Growth indices. **(A)** SPAD values. **(B)** Shoot FW. **(C)** Root FW. **(D)** Plasma membrane permeability. Perpendicular bars display averages ± SD (n = 6). Lower case letters above each bar present significant changes at *p* < 0.05 (Duncan’s multiple range test). DPF stands for days post flooding.

The impacts of flooding on chlorophyll content and other parameters (shoot and root fresh weights) were measured in the leaves of triploid (3X) and diploid (2X) watermelon varieties. The results suggest that there were no significant differences in the SPAD values in the 2X and 3X watermelon leaves at 0 days post flooding, which was considered as the control (**Figure 1A**). However, a decrease in the chlorophyll content was observed in both 2X and 3X watermelon leaves at 3 days, 5 days, and 7 days post flooding, and the decrease was significant in the 2X watermelon leaves. Additionally, the 3X watermelon showed higher shoot and root fresh weights compared to the 2X watermelon at all the time points in response to flooding (**Figures 1B, C**). The plasma membrane permeability was measured to assess the damage caused by flooding, and the results showed that at the early stages of flooding (0- and 3-days post flooding), the plasma membrane permeability was lower in both 2X and 3X watermelon leaves (**Figure 1D**). However, the 2X watermelon leaves had higher PMP compared to the 3X watermelon leaves at 5- and 7-days post flooding, indicating that the flooding caused more damage to the 2X watermelon as compared to the 3X watermelon. This suggests that the 3X watermelon was more tolerant to flooding compared to the 2X watermelon.

### 3.2 Fluctuations in O_2·−_ production rate, H_2_O_2_, as well as MDA contents

**Figure 2:**
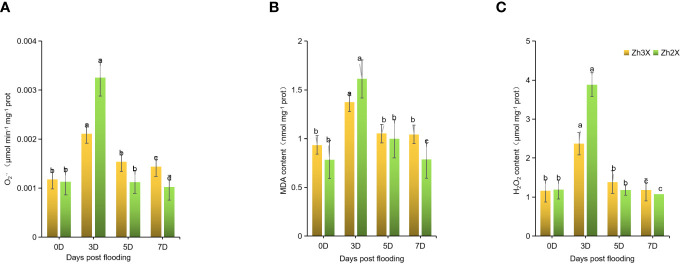
Fluctuation in ${\text{O}}_{2^{.-}}$ Production rate, H_2_O_2_, and MDA Contents. **(A)** ${\text{O}}_{2^{.-}}$ Production rate. **(B)** MDA content. **(C)** H_2_O_2_ content. Perpendicular bars display averages ± SD (n = 6). Lower case letters above each bar present significant changes at p < 0.05 (Duncan’s multiple range test).

Flooding impacts on the leaves of 2X and 3X watermelon were determined by recording variations in ${\text{O}}_{2^{.-}}$ , MDA and H_2_O_2_ contents. Current results suggests that no significant differences were observed at 0 days post flooding. However, at 3 days post flooding an increase in ${\text{O}}_{2^{.-}}$ production rate was recorded in 3X as compared to 2x watermelon leaves (**Figure 2A**). However, at 5- and 7-days post flooding it was observed that the ${\text{O}}_{2^{.-}}$ production rate remains stable in 3X watermelon leaves but higher ${\text{O}}_{2^{.-}}$ production rate in 2X watermelon leaves was observed. Similar trend in the MDA and H_2_O_2_ contents was observed in 2X and 3X watermelon leaves (**Figures 2B, C**). At 0 days post flooding no significant differences were observed in 2X and 3X watermelon whereas, a sharp increment in the activities of MDA as well as H_2_O_2_ were observed in 3X versus 2X. Whereas, at later stages of flooding stress the contents of MDA and H_2_O_2_ were recorded lower in 3X then 2X.

### 3.3 Variations in SOD, POD as well as CAT in response to flooding

**Figure 3:**
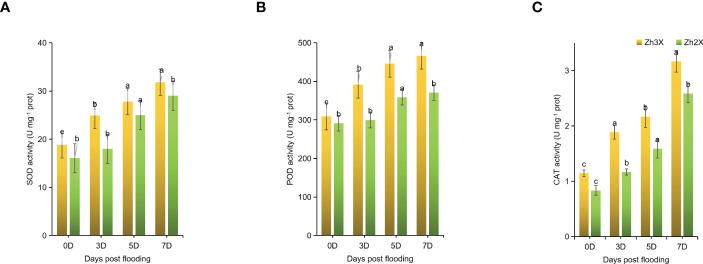
Fluctuation in the activities of SOD, POD, and CAT. **(A)** SOD, **(B)** POD), and **(C)** CAT. Perpendicular bars display averages ± SD (n = 6). Lower case letters above each bar present significant changes at *p* < 0.05 (Duncan’s multiple range test).

Accumulation of ROS in plants under stress leads to severe damages. For the eradication of these produced ROS antioxidant enzymes are required. Herein, the estimation of some antioxidants was performed in 2X and 3X watermelon leaves in response to flooding at different time points. Currently, it no significant increment in the activities of SOD, POD as well as CAT were observed at 0 days post flooding (**Figures 3A–C**). Whereas, at later stages of flooding i.e., 3-, 5- and 7-days post flooding a noticeable increment in SOD, POD as well as CAT were observed. Results indicated that the mentioned activities were significantly higher in 2X as compared to 3X watermelon leaves. Hence, it can be concluded that the antioxidant enzyme activities in 2X were higher as compared to 3X thus increasing the ability of 3X plants to cope waterlogging stress.

### 3.4 Fluctuations in non-enzymatic antioxidants activities

**Figure 4:**
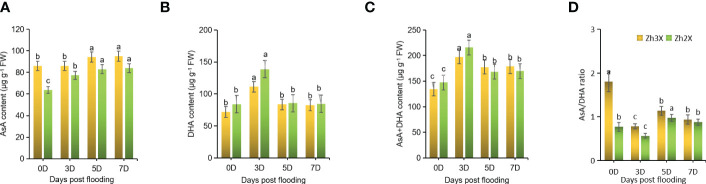
Fluctuations in Non-enzymatic Antioxidants. **(A)** AsA, **(B)** DHA, **(C)** AsA+DHA and **(D)** AsA/DHA ratio. Perpendicular bars display averages ± SD (n = 6). Lower case letters above each bar present significant changes at *p* < 0.05 (Duncan’s multiple range test).

In order to understand the role of non-enzymatic antioxidants (AsA and DHA) in building tolerance to flooding in watermelon, the levels of AsA, AsA+DHA, and AsA/DHA were measured. At 0 days post-flooding, no significant differences were found in the levels of AsA between 2X and 3X watermelon leaves. However, at later stages of flooding, an increase in the levels of AsA was observed in both 2X and 3X. Overall, 3X watermelon showed higher levels of AsA compared to 2X (**Figure 4A**). Additionally, 3X watermelon had higher levels of DHA (**Figure 4B**) and AsA+DHA as compared to 2X, but a notable decline in the AsA/DHA ratio was seen in both 2X and 3X at later stages of flooding compared to 0 days post-flooding (**Figures 4C, D**). The elevated levels of these non-enzymatic antioxidants make 3X watermelon more resistant to flooding.

### 3.5 Fluctuations in ROS scavengers (GSH, GSSG, GSH+GSSG, and GSH/GSSG ratio)

**Figure 5:**
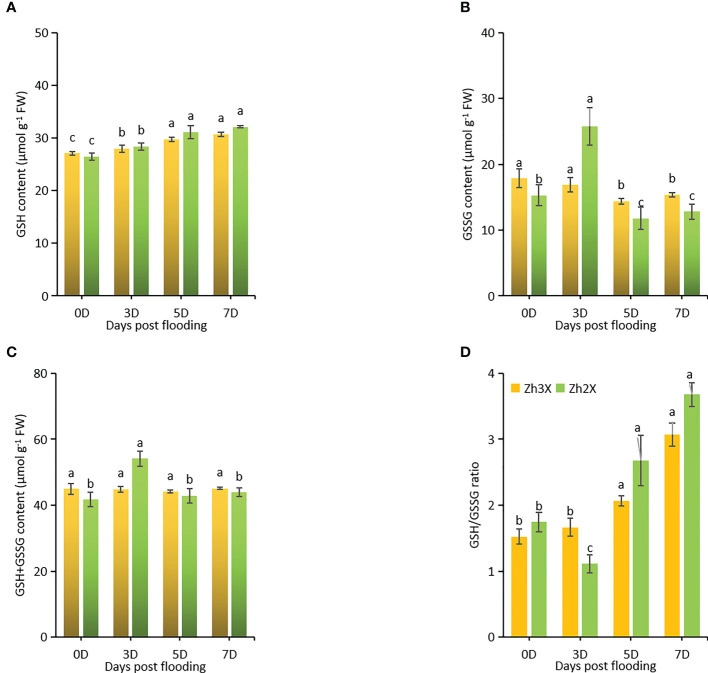
Fluctuations in the activities of ROS scavengers. **(A)** GSH, **(B)** GSSG, **(C)** GSH+GSSG, and **(D)** GSH/GSSG ratio. Perpendicular bars display averages ± SD (n = 6). Lower case letters above each bar present significant changes at *p* < 0.05 (Duncan’s multiple range test).

ROS scavengers like GSH, GSSG are crucial for plants to develop abiotic stress tolerance. Here, GSH and GSSG were estimated under flooding to assess their roles to boost flooding tolerance. Current results suggests that GSH levels were lower at 0 days post flooding in both 2X and 3X watermelon as compared to 3-, 5- and 7-days post flooding. However, a slightly higher GSH level was recorded in 3X as compared to 2X watermelon. Interestingly, higher GSSG levels were observed in 2X at 5 days post flooding as compared to 3X (**Figures 5A, B**). Increment of GSH+GSSG was recorded in 3X in under flooding (**Figure 5C**). Flooding exerted no significant effects on 3X as compared to 2X, thus GSH/GSSG ratios were higher in 3X as compared to 2X specifically at 7 days post flooding (**Figure 5D**).

### 3.6 Measurement of Ethylene production in response to flooding

**Figure 6:**
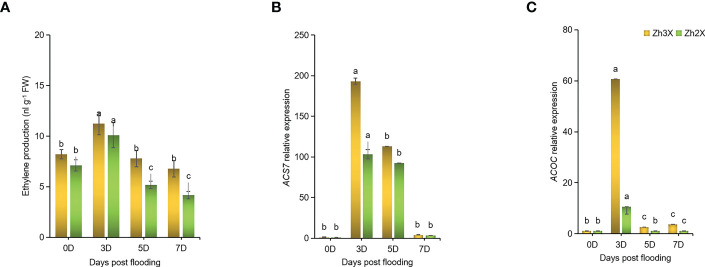
Fluctuations in Ethylene Biosynthesis in response to flooding. **(A)** Production of ethylene under flooding at different days post flooding. **(B)** Expression patterns of *WmACS7* as well as **(C)** *WmACOC* in watermelon leaves under flooding. Perpendicular bars display averages ± SD (n = 6). Lower case letters above each bar present significant changes at *p* < 0.05 (Duncan’s multiple range test).

The role of ethylene in adapting plants to flooding and regulating signals was studied. The production of ethylene was found to increase with time after flooding (**Figure 6A**). The effect of flooding on ethylene production was compared in 2X and 3X watermelon leaves. Initially, at 0 days post flooding, lower levels of ethylene were recorded in both types of watermelon. However, at later stages of flooding, the production of ethylene was found to be higher in 2X watermelon compared to 3X. The highest level of ethylene production was recorded at 5 days post flooding. Additionally, the expression of *WmACS6* and *WmACOC* genes was found to be higher at 5 days post flooding in 3X compared to 2X watermelon leaves (**Figures 6B, C**).

### 3.7 Distribution of detected metabolites in 2X and 3X watermelon leaves

**Figure 7:**
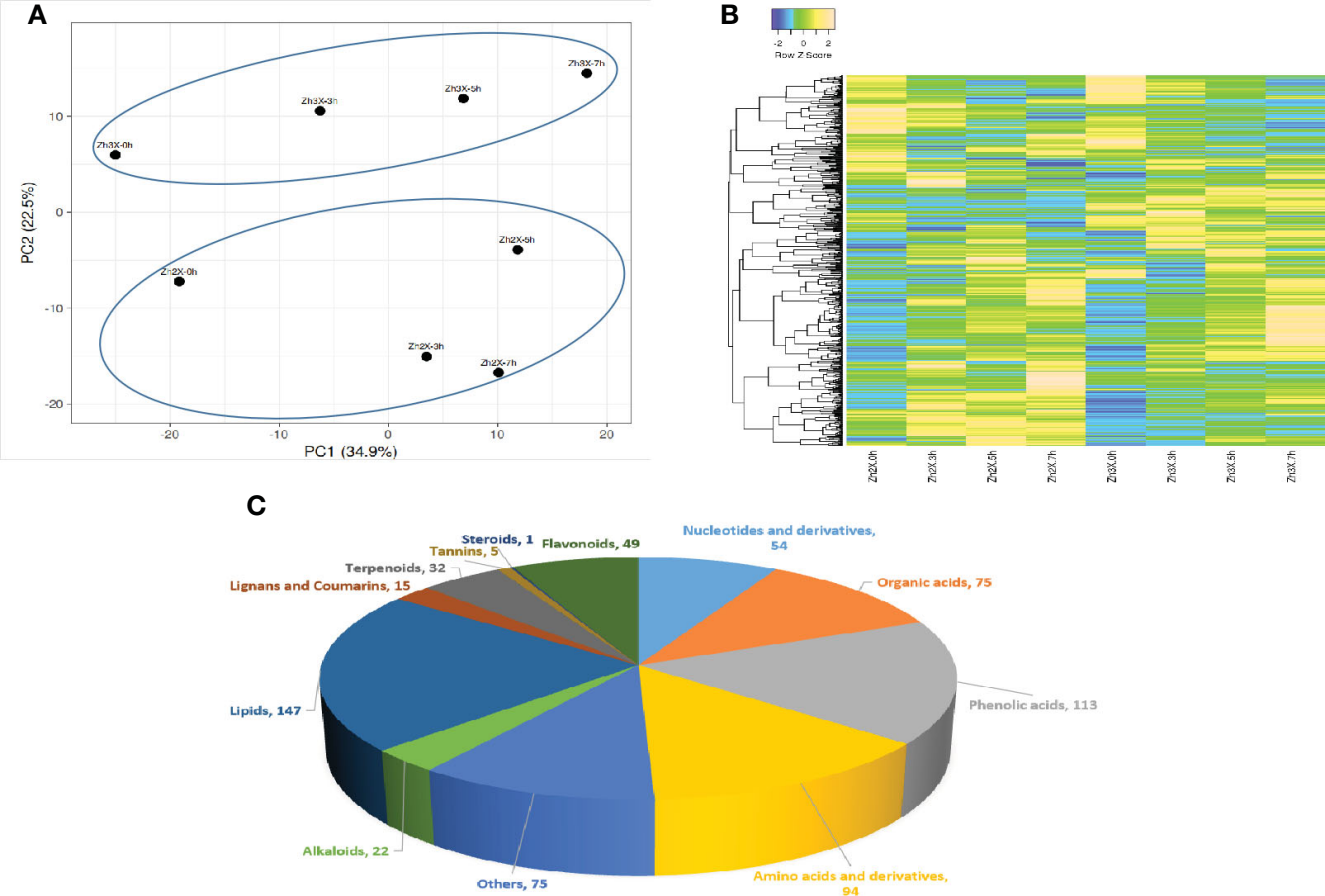
Cataloguing of metabolites in 2X and 3X watermelon leaves under flooding stress. **(A)** PCA plot presenting metabolic variations in 2X and 3X watermelon leaves at different days post flooding. **(B)** Heatmap illustrating metabolic contents among 2X and 3X watermelon leaves under flooding stress. **(C)** Detected metabolites are categorized into diverse groups on annotation basis.

The leaves of 2X and 3X watermelon plants were gathered at 0, 3, 5 and 7 DPF for metabolites quantification. Totally, 682 metabolites were detected and annotated in both 2X and 3X watermelon leaves in response to flooding stress. Up and down regulated metabolites are represented in a tabular form as **Supplementary Table S3**. Principal component analysis (PCA) explained the data variability suggesting that current data is dependable and can be processed further (**Figure 7A**). A heatmap was generated to represent the expression patterns of all the detected metabolites in 2X and 3X watermelon leaves at different days after flooding stress (**Figure 7B**). Among the annotated metabolites 86% were categorized into various groups whilst 14% were grouped into the category of others (**Figure 7C**). Current data shows that the expression patterns of metabolites belonging to various categories are also different among 2X and 3X watermelon. Overall lipids share 21%, followed by phenolic acids (17%), amino acids and derivatives (14%), organic acids (11%), Nucleotides and derivatives (8%), flavonoids (7%), terpenoids (5%), alkaloids (3%), steroids (1%), lignin and coumarins (2%) and tannins (1%) respectively.

### 3.8 KEGG enrichment of detected metabolites in 2X and 3X watermelon leaves at different days post flooding

**Figure 8:**
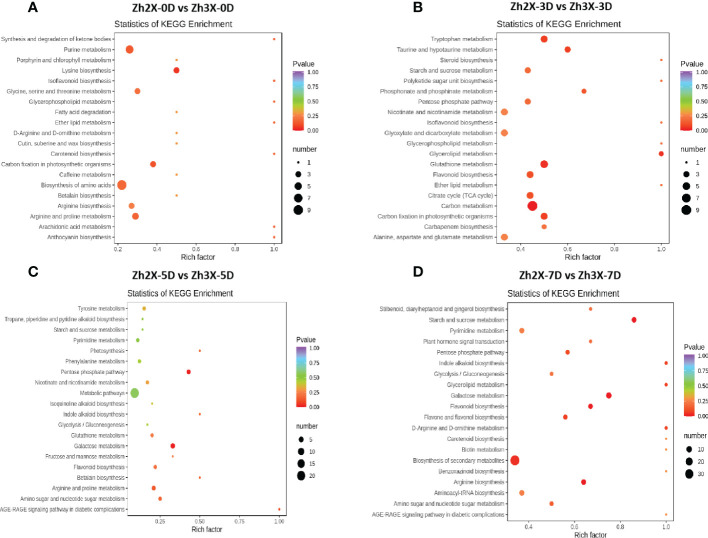
KEGG enrichment of detected metabolites. **(A)** Zh2X-0D vs Zh3X-0D, **(B)** Zh2X-3D vs Zh3X-3D **(C)** Zh2X-5D vs Zh3X-5D and **(D)** Zh2X-7D vs Zh3X-7D.

According to the KEGG enrichment current results suggested that when 2X and 3X watermelon were compared at 0 days post flooding, we observed that the biosynthesis of amino acids, purine metabolism, arginine as well as purine metabolic pathways were highly enriched (**Figure 8A**). Similarly, at 3 days post flooding carbon metabolism, tryptophan metabolism, flavonoid biosynthesis pathways were enriched (**Figure 8B**). At 5 days post flooding we observed that galactose metabolism, arginine, and purine biosynthetic pathways were highly enriched (**Figure 8C**). Moreover, at 7 days post flooding biosynthesis of secondary metabolites was highly enriched (**Figure 8D**).

### 3.9 Identification of top foldchange metabolites in each comparison group

**Figure 9:**
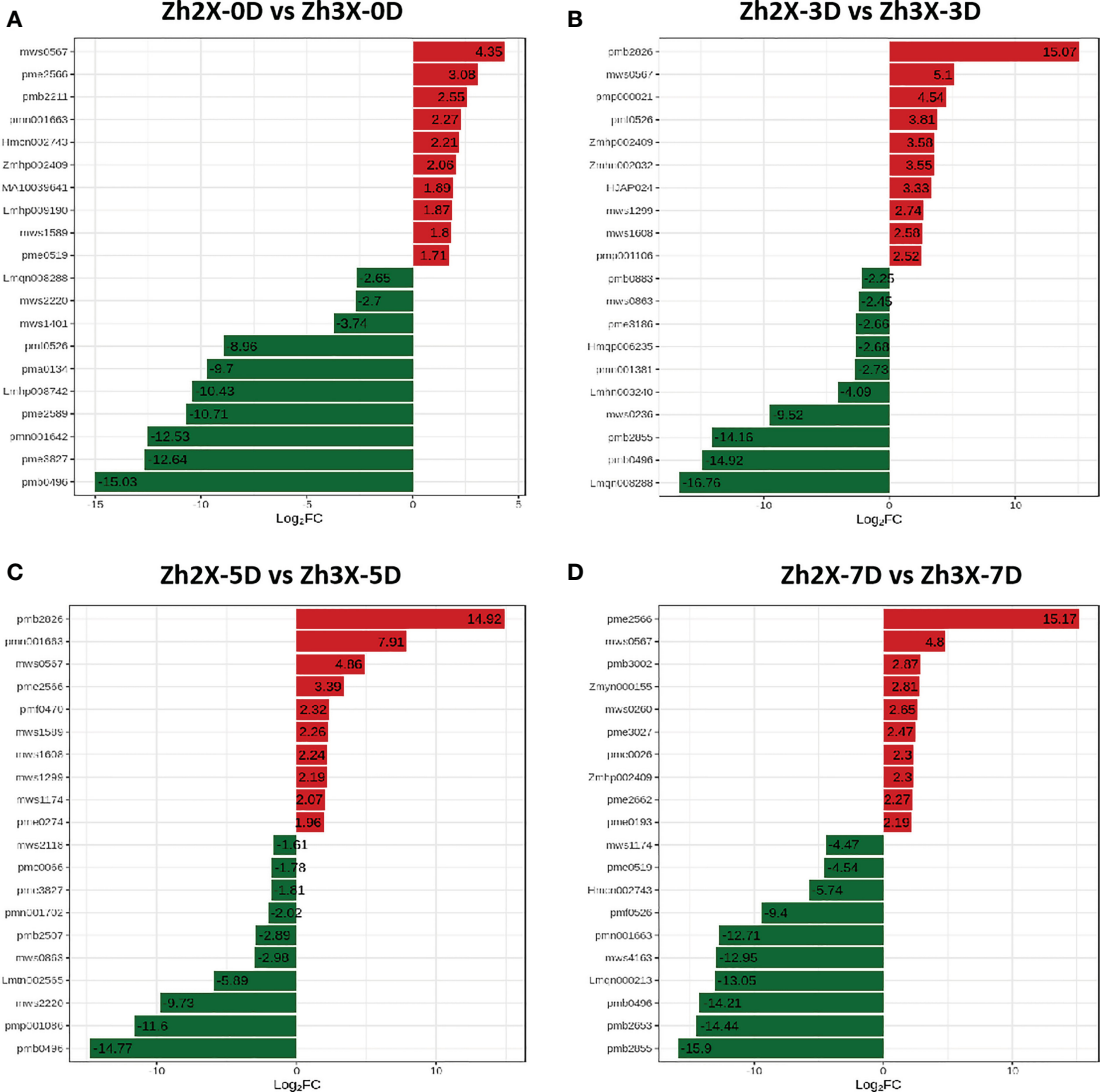
TopFC metabolites in each comparison group at different days post flooding. **(A)** Zh2X-0D vs 347 Zh3X-0D, **(B)** Zh2X-3D vs Zh3X-3D, **(C)** Zh2X-5D vs Zh3X-5D, **(D)** Zh2X-7D vs Zh3X-7D.

To better understand the metabolic changes that occur in response to flooding, we analyzed annotated metabolites to identify those that were significantly increased in both 2X and 3X watermelon leaves at various time points post-flooding. The comparison between 2x-0D (diploid leaves at 0 days after flooding) and 3X-0D (triploid leaves at 0 days after flooding) showed that mws0567 (4-Guanidinobutyric acid), pme2566 (5-L-Glutamyl-L-amino acid), Pmb2211 (Cocamidopropyl betaine), pmn001663 (Syringaresinol), Hmcn002743 (Lirioresinol A), Zmhp002409 (Isoluteolin-6,8-di-C-glucoside), MA10039641 (Lactobiose), Lmhp009190 (2-Linoleoylglycerol-1,3-di-O-glucoside*), mws1589 (D-Panose) and pme0519 (D-Sucrose) were up-regulated with high log2FC values (**Figure 9A**). Similarly the comparison group 2x-03 (diploid at three days post flooding) versus 3X-3D (triploid at three days post flooding) we observed that pmb2826 (L-Citramalic acid), mws0567 (4-Guanidinobutyric acid), pmp000021 (Isooxypeucedanine), pmf0526 (Isoimperatorin), Zmhp002409 (Isoluteolin-6,8-di-C-glucoside), Zmhn002032 (Glucosyl-caffeoyl-glucosyl)-4-hydroxybenzyl alcohol), HJAP024 (Kaempferol-6,8-di-C-glucoside), mws1299 (Luteolin-8-C-glucoside (Orientin)*), mws1608 (Luteolin-6-C-glucoside (Isoorientin)) and pmp001106 (Vitexin-2’’-O-glucoside) were highly upregulated with top log2FC values (**Figure 9B**). Moreover, in the comparison group 2x-5D (diploid at five days post flooding) versus 3X-5D (triploid at five days post flooding) we found that pmb2826 (L-Citramalic acid), pmn001663 (Syringaresinol), mws0567 (4-Guanidinobutyric acid), pme2566 (5-L-Glutamyl-L-amino acid), pmf0470 (6-Aminocaproic acid), mws1608 (Luteolin-6-C-glucoside (Isoorientin)), mws1299 (Luteolin-8-C-glucoside (Orientin)*), mws1174 (3-O-Acetylpinobanksin), pme0274 (6-Aminocaproic acid) were found to be highly upregulated with highest log2FC values (**Figure 9C**). In case of comparison group 2x-07 (diploid at seven days post flooding) versus 3X-7D (triploid at seven days post flooding), pme2566 (5-L-Glutamyl-L-amino acid), mws0567 (4-Guanidinobutyric acid), pmb3002 (Chrysoeriol-7-O-rutinoside), Zmyn000155 (N-α-Acetyl-L-ornithine), mws0260 (L-Arginine), pme3027 (N-Acetyl-L-Cysteine), pme0026 (L-Lysine), Zmhp002409 (Isoluteolin-6,8-di-C-glucoside), pme2662 (Vitamin D3 (Cholecalciferol)) and pme0193 (L-Glutamine) had the highest log2FC values and are highly upregulated (**Figure 9D**). Interestingly, we noticed that mws0567 (4-Guanidinobutyric acid) was found to be highly upregulated in all the mentioned comparison groups and it belongs to the class organic acids. Similarly, there were some other highly upregulated metabolites that were common among at least 2 comparison groups such as pmb2826 (L-Citramalic acid) present in 2X-3D vs 3X-3D and 2X-5D vs 3X-5D. Most upregulated metabolites belong to class flavonoids, organic acids, amino acids and derivatives and phenols respectively. It can be assumed that metabolites belonging to the mentioned classes play a significant role in flooding stress tolerance.

### 3.10 Variations in top foldchange metabolites among 2X and 3X wateremlon

**Figure 10:**
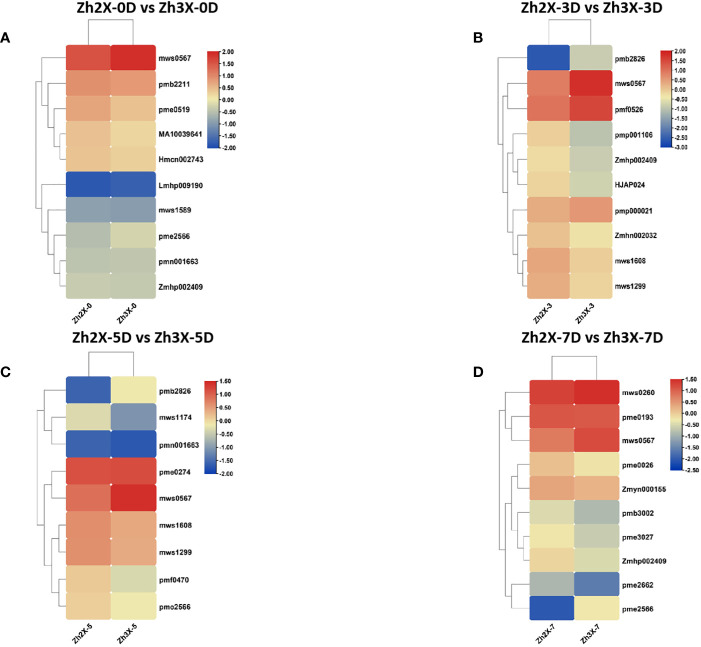
Variations in Top Foldchange metabolites among 2X and 3X watermelon. **(A)** Expression of metabolites in Zh2X-0D vs Zh3X-0D comparison group**(B)** Expression of metabolites in Zh2X-3D vs Zh3X-3D comparison group **(C)** Expression of metabolites in Zh2X-5D vs Zh3X-5D comparison group, and **(D)** Expression of metabolites in Zh2X-7D vs Zh3X-7D comparison group.

Heatmaps were drawn to represent the expression patterns of highly upregulated metabolites in both 2X and 3X watermelon leaves in response to flooding. At 0 days past flooding it was observed that the content of mws0567 (4-Guanidinobutyric acid) was higher in 3X watermelon leaves then 2X (**Figure 10A**). At three days post flooding results indicated that mws0567 (4-Guanidinobutyric acid) and pmf0526 (Isoimperatorin) have higher contents in 3X watermelon as compared to 2X watermelon (**Figure 10B**). Moreover at 5 days post flooding the contents of mws0567 (4-Guanidinobutyric acid) higher in 3X watermelon leaves then 2X (**Figure 10C**). Moreover, at seven days post flooding the contents of mws0567 (4-Guanidinobutyric acid) and mws0260 (L-Arginine) were higher in 3X watermelon leaves then 2X (**Figure 10D**). From the above results it can be suggested that mws0567 (4-Guanidinobutyric acid) belonging to class organic acids might be the candidate metabolite that is responsible for flooding tolerance in watermelon and its higher expressions in triploid watermelon leaves makes it more tolerant to flooding as compared to diploid watermelon. Overall. It can be assumed that triploid watermelon are much more tolerant to diploid watermelon.

## 4 Discussion

The impacts of climate change on crop production have been significant worldwide (Schmidhuber and Tubiello, 2007). Plants face numerous challenges in the form of biotic and abiotic stress throughout their growth cycle (Savvides et al., 2016). Flooding is a prevalent abiotic stress that poses a significant threat to the production of watermelon, a horticultural crop (Onaga and Wydra, 2016; Parmar et al., 2017; Zhang et al., 2022). Although plants have evolved strategies to cope with biotic and abiotic stress, not all of them are tolerant to adverse environmental changes, especially flooding (Gull et al., 2019; Cao et al., 2022). Therefore, current research is aimed at developing plant resources that are resistant to flooding and have good yield and production.

Recent advancements in technology have made it possible to delve deeper into the inner workings of plants in response to various stressors (Chaves et al., 2003; Yan et al., 2022). Transcriptomics provides valuable insights into gene regulation at specific time points, allowing us to determine the role of genes based on whether they are up or down-regulated (Boeck et al., 2016; Wang et al., 2022). Metabolites also play a critical role in plant tolerance to both biotic and abiotic stresses (Romero et al., 2018; Jan et al., 2021). They serve as the first line of defense in response to external stimuli, and metabolomics has become an important tool for detecting and characterizing significant metabolites in response to various stressors (Chen et al., 2013; Mashabela et al., 2022). These technological advancements in transcriptomics and metabolomics can greatly aid in uncovering previously unknown facts.

Flooding has a major impact on plant growth and development, leading to stunted and slower growth (Joshi, 2018). This stressor affects both the phenotype and physiology of plants, resulting in inadequate nutrient uptake and chlorosis, ultimately leading to plant death (Arbona et al., 2008; De Castro et al., 2022). In this study, we aim to examine how diploid and triploid watermelon respond to flooding and the resulting changes in plant physiology and biochemistry.

SPAD meter was used to estimate the chlorophyll content in 2X and 3X watermelon leaves in response to flooding at 0, 3, 5 and 7 DPF. Higher chlorophyll contents were observed in 3X watermelon. Present results suggests that the chlorophyll contents in response to flooding reduce significantly. Our results agree with previous report which suggests that flooding results in lower chlorophyll contents but triploid watermelon has more chlorophyll as compared to diploid watermelon (Yan et al., 1996; Choudhary and Padmanabhan, 2021; He et al., 2022). Similarly, root fresh weight and shoot fresh weight are important in terms of a plant’s response to stress (Pires et al., 2015; Gálvez et al., 2021; Zhang et al., 2021). Our results indicated that 3X watermelon possess higher root and shoot fresh weights as compared to 2X watermelon plants (Garg et al., 2002; Kartik et al., 2021). Cell damage and plasma membrane permeability are the indicators of damage that occur due to flooding (Yeung et al., 2019). Current results indicate that 3X watermelon have lower plasma membrane permeability as compared to 2X watermelon.

In the current research work we estimated the O_2_ production rate, MDA and H_2_O_2_ contents in response to flooding at different time intervals (Yan et al., 1996; Seymen, 2021; Teoh et al., 2022). The said parameters are important to understand the strength of plants to cope flooding. Our results indicated that highest O_2_ production rate, MDA and H_2_O_2_ contents were observed at 3 days post flooding but 3X have lower values as compared to 2X. Our findings suggests that overall 3X watermelon had lower O_2_ production rate, MDA and H_2_O_2_ contents as compared to 2X watermelon in response to flooding. Flooding causes larger accumulations of ROS therefore, worsening oxidative damage to cellular metabolites including proteins, lipids, as well as nucleic acids. Exorbitant ROS in plant cells cause lipid peroxidation although at the same time increasing ${\text{O}}_{2^{-}}$ , H_2_O_2,_ and MDA levels. which is in agreement with previous studies (Yetisir et al., 2006; He et al., 2022).

Our findings indicate that the activity levels of antioxidant enzymes, such as SOD, POD, and CAT, play a crucial role in plant adaptation to flooding. Our results show that 3X watermelon exhibited higher levels of these enzymes compared to 2X watermelon at 0-, 3-, 5-, and 7-days post-flooding (DPF). Previous studies have also highlighted the importance of higher antioxidant levels in stress tolerance. Flooding causes both physiological and phenotypic damage to plants, hindering growth by reducing the uptake of essential nutrients. This leads to nutritional imbalances, stunted root and shoot development, chlorosis, and eventually, plant death (Arbona et al., 2008). Higher levels of DHA and AsA+DHA were noticed in 3X then 2X, however a significant decline in AsA/DHA ratio was observed in 3X as well as 2X at later stages of flooding as compared to 0 days post flooding.

Nonenzymatic antioxidants, including AsA are crucial to protect cells against ROS thus by maintaining the redox balance under environmental stresses. APX as well as DHAR are the two main spanner enzymes involved in AsA-GSH cycle. Studies showed that APX is the most important scavenging enzyme ensuring the scavenging capability of reactive oxygen species (Czarnocka and Karpiński, 2018). Furthermore, it has been previously reported that for mitigating and enhancing stress tolerance AsA/DHA ratios are far more important than AsA contents (Szalai et al., 2009; Yu et al., 2021; Tai et al., 2022). Current results revealed lower AsA/DHA as well as GSH/GSSG ratios in 3X watermelon as compared to 2X. Therefore, our findings suggest that 3X has an improved antioxidant capability by modifying non-enzymatic antioxidant system to preserve the redox balance (Sang et al., 2016).

Phenotypic as well as biochemical symptoms occurs due to flooding. Moreover, it has been reported earlier that flooding also leads to ethylene production (Casierra-Posada and Cutler, 2017). Currently we observed a higher production of ethylene in 2X watermelon as compared to 3X watermelon as previously reported in perennial pepper weed (Chen, 2002), avocado, as well as cotton (Zhang et al., 2019) triggering leaf senescence (Zhang et al., 2019). Ethylene can exacerbate the effects caused by abiotic stresses, given the fact that the harmful effect of flooding might be alleviated by lowering concentrations of endogenous ethylene (Najeeb et al., 2015). Herein, reduced ethylene production was observed in 3X watermelon as compared to 2X watermelon.

Ethylene production depends on the expressions of ACS and ACO and the influence of other external and internal factors. In roots high ACC is produced which is then transferred to shoots where it is converted to ethylene *via* ACO (Sasidharan and Voesenek, 2015). Flooding causes a higher expression of *ACS6* as well as *ACOC* n 3X watermelon plants was observed as compared to 2X along with increased ethylene production.

Metabolites are integral components in a plant’s stress response. Our study employed a UPLC-ESI-MS/MS-based methodology for quantifying metabolites and ultimately detected a total of 682 unique metabolites. The analysis revealed that the metabolite, 4-Guanidinobutyric acid (mws0567), which falls under the category of organic acids, may play a crucial role in promoting flood tolerance in watermelon plants. Furthermore, the results indicate that the expression of this metabolite was higher in triploid watermelon (3X) as compared to diploid watermelon (2X), thereby conferring superior tolerance to flooding stress.

**Figure 11:**
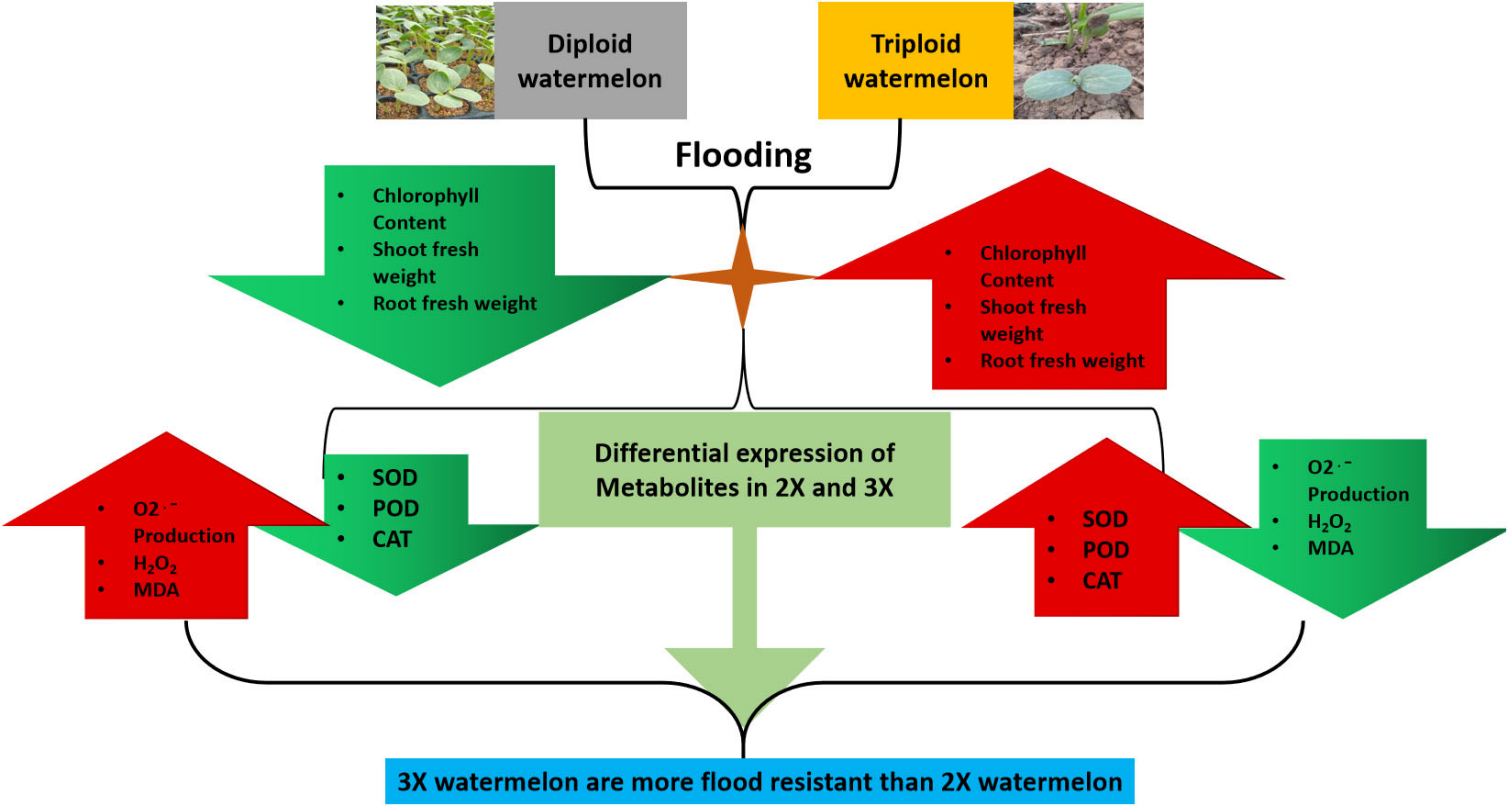
A schematic representation of overall phenological, physiological and biochemical variations among 2X and 3X watermelon leaves in response to flooding at different time points.

This study sheds light on the complex interplay between metabolites and flood tolerance in watermelon and highlights the potential of UPLC-ESI-MS/MS technology in exploring the molecular underpinnings of plant stress response (**Figure 11**).

## 5 Conclusion

The present study aimed to evaluate the physiological, biochemical, and metabolic responses of diploid and triploid watermelon under flooding stress conditions. The results indicated a lower chlorophyll content and reduced shoot and root fresh weights in diploid watermelon compared to the triploid counterpart. The activities of antioxidants including superoxide dismutase (SOD), peroxidase (POD), and catalase (CAT) were found to be elevated in triploid watermelon, accompanied by a decrease in oxygen production rates, malondialdehyde (MDA) and hydrogen peroxide (H2O2) levels. Additionally, higher ethylene production was observed in triploid watermelon leaves in response to flooding. A UPLC-ESI-MS/MS-based metabolomics approach was applied and a total of 682 metabolites were detected, among which 4-Guanidinobutyric acid, an organic acid, emerged as a potential candidate metabolite responsible for the tolerance to flooding in watermelon, exhibiting higher expression levels in triploid watermelon. These results suggest that triploid watermelons may exhibit increased tolerance to flooding stress compared to diploid watermelons. This research provides important insights into the responses of watermelon to flooding and further molecular and genetic studies are necessary to gain a deeper understanding of the underlying mechanisms of flooding tolerance in this crop.

## Data availability statement

The datasets presented in this study can be found in online repositories. The names of the repository/repositories and accession number(s) can be found in the article/**Supplementary Material**.

## Author contributions

XS, NH, and WL, complete the experimental design. WL, HZ and XL were responsible for the collection of experimental materials and samples. NH, PY, WW and YX, were responsible for the completion of the experiment, NH was mainly responsible for the manuscript writing, MU, RB and CG were mainly responsible for the manuscript proofreading. All authors contributed to the article and approved the submitted version.

## Supplementary material

Supplementary Table 1 List of primers used for qRT-PCR.
