# Supplementary material for: Physiological, biochemical, and metabolic changes in diploid and triploid watermelon leaves during flooding
Source: Front Plant Sci. 2023 Mar 9;14:1108795. doi: 10.3389/fpls.2023.1108795 (PMC10033695; doi:10.3389/fpls.2023.1108795)
Supplement: Supplementary Table 1 — List of primers used for qRT-PCR. [file Table_1.docx]

**Supplementary Table S1 Sample description and time points for flooding**

| **Name** | **Tissue** | **Sample Description** |
| --- | --- | --- |
| Zh2X-CK | Leaves | Diploid. 0 days after flooding |
| Zh3X-CK | Leaves | Triploid. 0 days after flooding |
| Zh2X-3 | Leaves | Diploid. 3 days after flooding |
| Zh3X-3 | Leaves | Triploid. 3 days after flooding |
| Zh2X-5 | Leaves | Diploid. 5 days after flooding |
| Zh3X-5 | Leaves | Triploid. 5 days after flooding |
| Zh2X-7 | Leaves | Diploid. 7 days after flooding |
| Zh3X-7 | Leaves | Triploid. 7 days after flooding |
