# Supplementary material for: Physiological, biochemical, and metabolic changes in diploid and triploid watermelon leaves during flooding
Source: Front Plant Sci. 2023 Mar 9;14:1108795. doi: 10.3389/fpls.2023.1108795 (PMC10033695; doi:10.3389/fpls.2023.1108795)
Supplement: Supplementary file 2 [file Table_2.docx]

**Supplementary Table S2** Number of up and down regulated metabolites in the leaves of diploid and triploid watermelon.

| **Comparison Group** | **Total number of significant metabolites** | **Up regulated** | **Down regulated** |
| --- | --- | --- | --- |
| **Zh2X-CK vs Zh3X-CK** | 111 | 51 | 60 |
| **Zh2X-3 vs Zh3X-3** | 152 | 53 | 99 |
| **Zh2X-5 vs Zh3X-5** | 78 | 37 | 41 |
| **Zh2X-7 vs Zh3X-7** | 194 | 82 | 112 |
